# Two-Stage Pediatric Ear Reconstruction Using Preserved Native Cartilage After a Dog Bite

**DOI:** 10.31486/toj.22.0045

**Published:** 2023

**Authors:** H. Harvak Hajebian, Salomon Puyana, Igor Burko, Michael T. Friel

**Affiliations:** ^1^ Department of Plastic and Reconstructive Surgery, Ochsner Clinic Foundation, New Orleans, LA; ^2^ The University of Queensland Medical School, Ochsner Clinical School, New Orleans, LA; ^3^ Division of Plastic Surgery, Department of Surgery, Tulane University School of Medicine, New Orleans, LA

**Keywords:** *Bites and stings*, *dogs*, *ear cartilage*, *pediatrics*, *reconstructive surgical procedures*, *wounds and injuries*

## Abstract

**Background:** A dog bite causing an auricular avulsion is a rare cause of an outer ear defect. By nature of the high-energy trauma inflicted by canine bites and the anatomic variability of the outer ear, no two such avulsion injuries are the same. If the native cartilage cannot be preserved after trauma, placement of a graft capable of forming grooves and ridges is required to reconstruct the complex anatomy of the outer ear. Such intricacies often make postoperative results cosmetically disappointing. In select cases, the native cartilaginous framework of the avulsed ear segment may be preserved and used in reconstruction.

**Case Report:** We report a case of a pediatric total auricular avulsion following a dog bite, reconstructed using prelaminated native ear cartilage.

**Conclusion:** After traumatic avulsion of the outer ear when native cartilage is preserved, effective reconstruction can be achieved using a 2-stage technique of native cartilage lamination via posterior auricular pocket formation and placement of a skin graft.

## INTRODUCTION

In the United States, reconstructive surgery for injuries caused by dog bites occurs at an incidence of 4.6 persons per 100,000 population.^1^ The most common anatomic site of injury is the upper extremity, followed by the lower extremity, with the majority of reported cases occurring in the pediatric population.^2^ While injuries affecting the extremities compose the majority of cases within the pediatric age group, dog bites affecting the face, head, and neck region occur most frequently in patients <6 years old.^2^ Reports of dog bites that result in a complete avulsion of an outer ear segment are rare, and a scarcity of published cases exists in the literature.^3-7^

In cases of auricular defects in which direct replantation is not feasible, surgical reconstruction of the missing region is required. The surgeon is tasked to create a model with grooves and ridges that is fixed and steady enough to project from the proximal auricular appendage while staying aligned with the angle of projection off the cranium. The outer ear's unique anatomic structure of cartilage covered by thin and pliable skin creates a significant challenge in achieving aesthetic results, and reconstructions are often cosmetically disappointing.^5,8-10^ Because of the complex anatomic design, a satisfactory outcome greatly depends on the type of surgical reconstruction performed. Small defects can be treated with skin grafting alone, while larger defects require replantation or formal ear reconstruction with cartilage grafts or prosthetic devices.^8-12^

The most common method for successful reconstruction is the pocket principle, a 2-stage approach that involves removing the skin from the outer ear cartilage and burying the cartilage in a subcutaneous pocket located in the posterior auricular space beneath a thick layer of skin and subcutaneous tissue.^13,14^ This technique provides a robust blood supply via the posterior auricular and superior temporal arteries and has been shown to be an effective method for providing the anterior skin surface for prelaminated cartilage grafts and supporting subsequent framework elevation.^5,9,13,14^

We present a case of a 2-stage auricular reconstruction using prelaminated native amputated cartilage via posterior auricular pocket formation and placement of a skin graft.

## CASE REPORT

An 11-year-old healthy male was transported to the emergency department (ED) after being bitten on the ear by his family's dog. The patient had an avulsion injury to the left auricle with complete amputation of the outer ear. The injury occurred approximately 2 hours before the patient arrived in the ED, and the patient's guardian conserved the amputated ear segment in a bag of ice immediately following the event. The dog was a Rhodesian ridgeback and was reportedly unprovoked at the time of attack. All the dog's vaccinations, including rabies, were confirmed to be up to date, and the patient had no other associated injuries.

**Figure 1.**
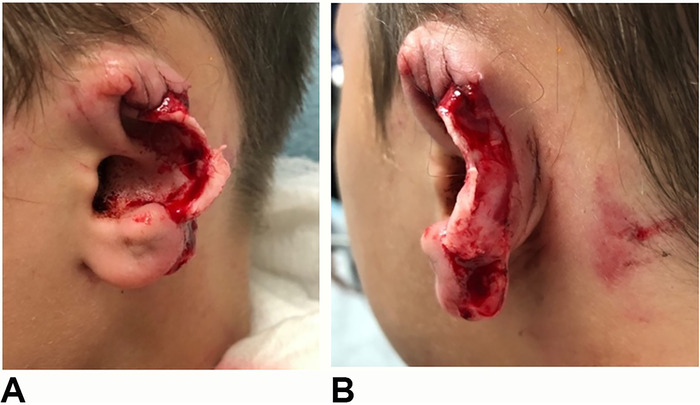
(A) Traumatic ear defect preserving the concha, ear lobe, and root of helix. (B) Posterior auricular view of ear defect.

**Figure 2.**
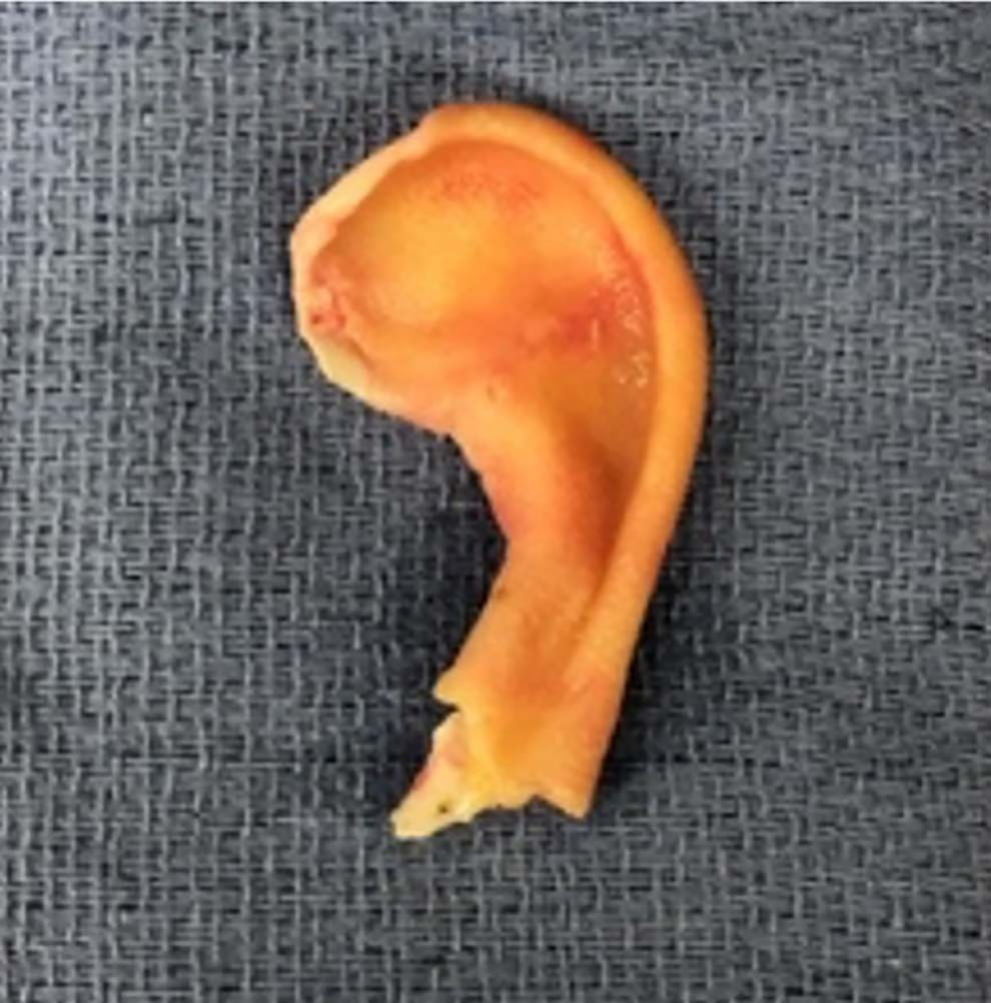
Anterior view of the amputated ear segment.

The part of the ear that remained on the child was the concha, the ear lobe, and the root of the helix (Figure 1). The amputated segment involved all of the helix, part of the antihelix, the superior limb of the triangular fossa, and the scapha fossa to the level of the concha (Figure 2).

**Figure 3.**
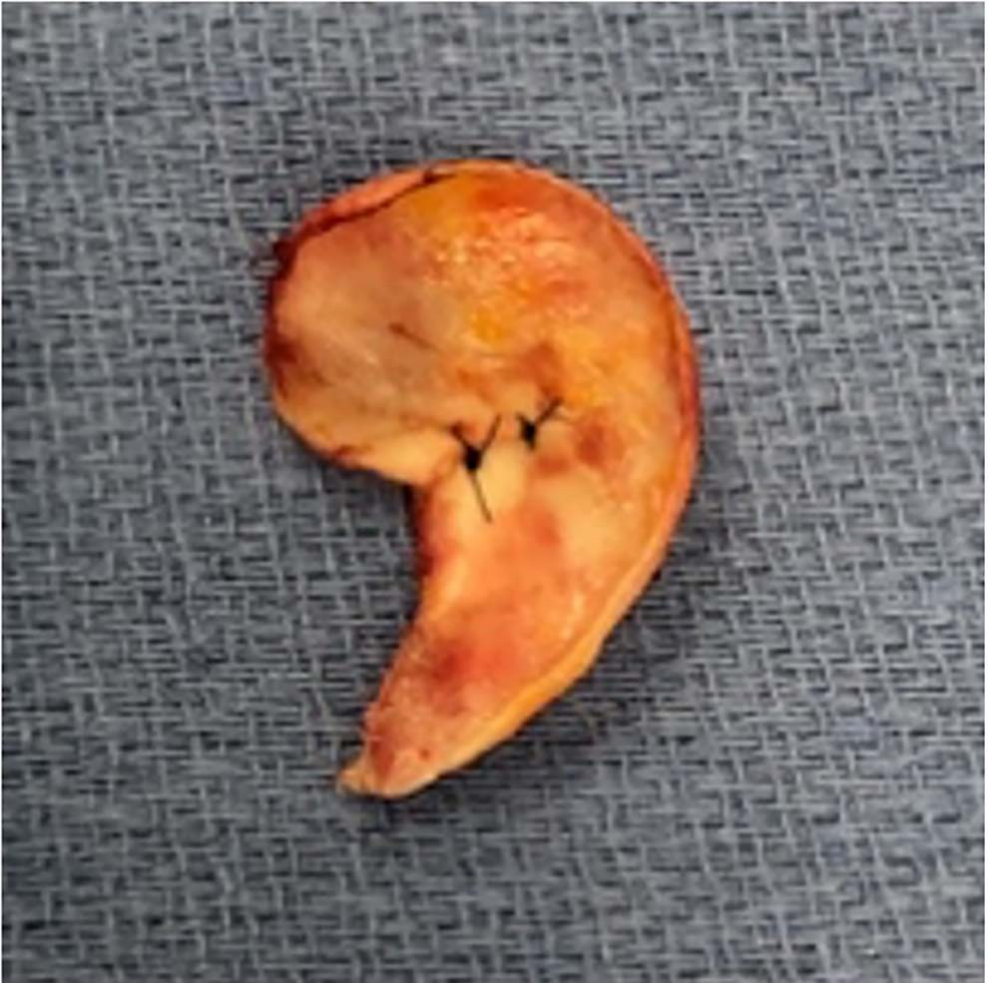
Preserved de-skinned amputated cartilage.

The amputated ear segment was examined under high-power magnification, and no appreciable targets for supermicrosurgery were identifiable for a possible arterial anastomosis, a finding consistent with the severe crushing and tearing nature of the injury. As a result, the decision was made to de-skin the amputated ear segment, leaving the perichondrium in place for lamination via posterior auricular pocket formation in the first stage of a 2-stage ear reconstruction (Figure 3).

**Figure 4.**
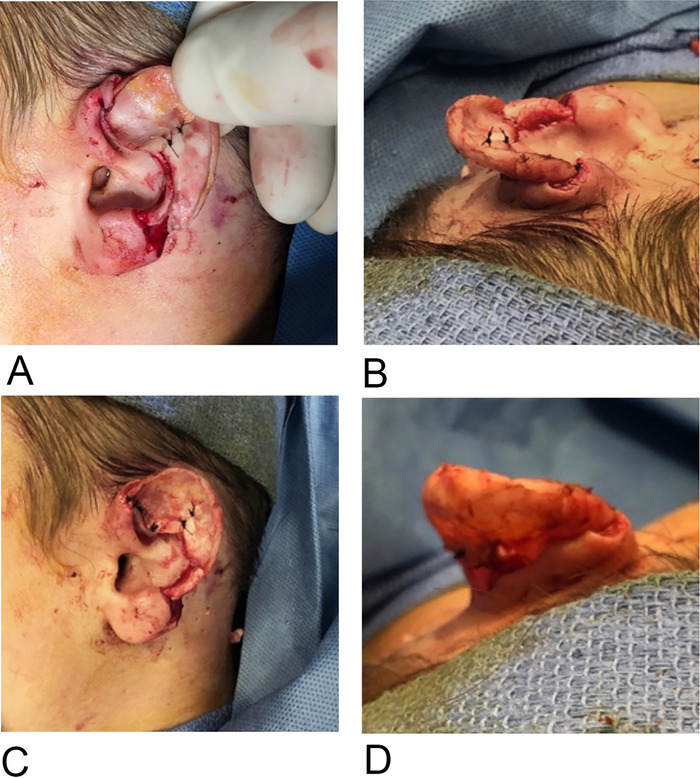
(A) Pre-pocket burial approximation of the reconstruction plan. (B) Superior and (C) anterior view of pre-pocket burial native cartilage approximation. (D) Posterior view of pre-pocket burial approximation, revealing the future defect that will require full-thickness skin grafting.

In the second stage, after integration and neovascularization of the native cartilage, the prelaminated framework would be elevated for replantation. The risk of the lamination process failing is high because of cartilage reabsorption; however, if lamination is successful, the native cartilage would remain perfused and eliminate the need for additional cartilage grafts (Figure 4).

### First-Stage Procedure: Posterior Auricular Pocket Formation

**Figure 5.**
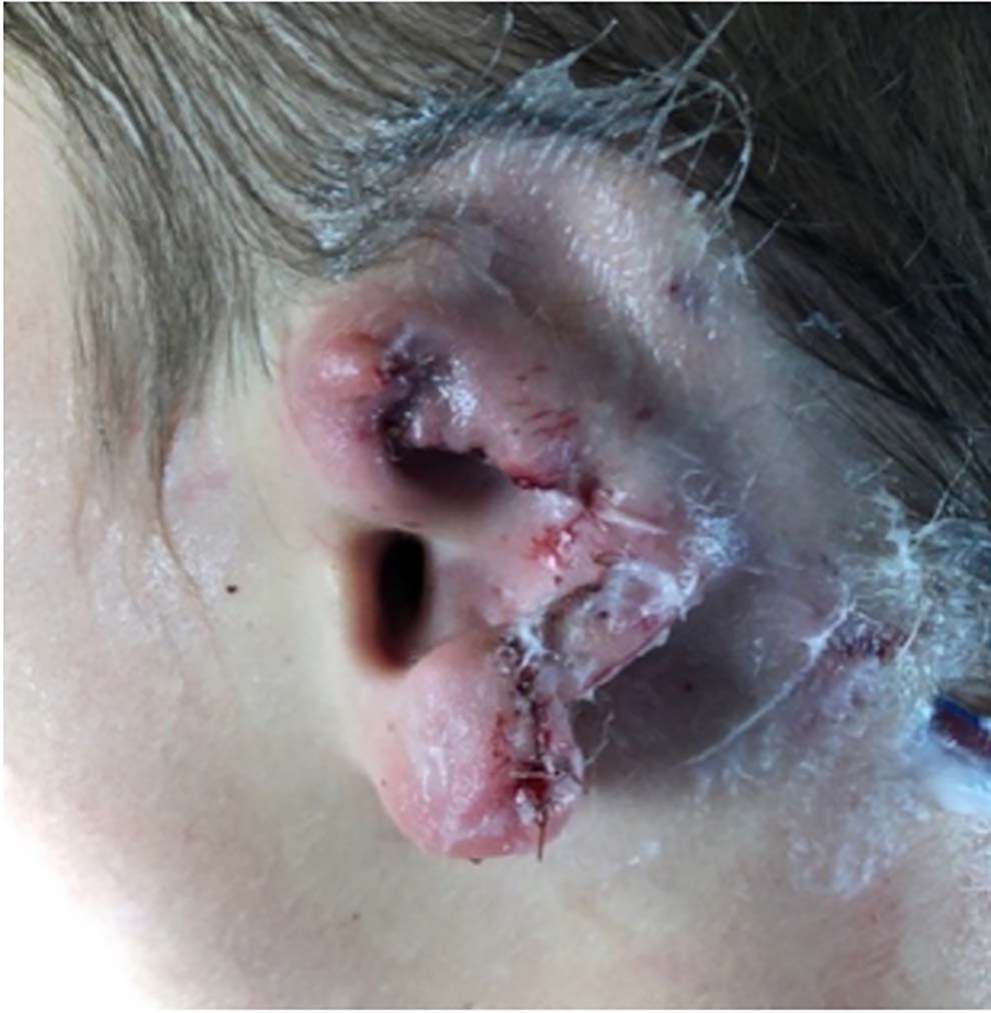
Postoperative result of stage 1: de-skinned amputated ear cartilage buried in posterior auricular pocket with native ear wound closure and bacitracin dressing.

With tenotomy scissors, the posterior auricular pocket was made from the retroauricular sulcus to the occipital scalp, taking care to leave the posterior auricular artery and nerve undisturbed. The dissection stayed lateral and inferior, thereby allowing for the temporalis fascia to be harvested later if needed. The pocket measured 5 cm × 6 cm. With the pocket developed, the cartilage of the amputated ear segment was trimmed and approximated to the native ear stump at the amputation site with nylon sutures. The ear construct was then buried for lamination under the superficial posterior auricular skin flap. A vacutainer drain was placed at the posterior aspect of the posterior auricular pocket and remained in place for 10 days. The skin edges were approximated with chromic sutures (Figure 5). The ear was covered with a Glasscock dressing.

### Second-Stage Procedure: Native Cartilage Framework Elevation and Placement of a Skin Graft

**Figure 6.**
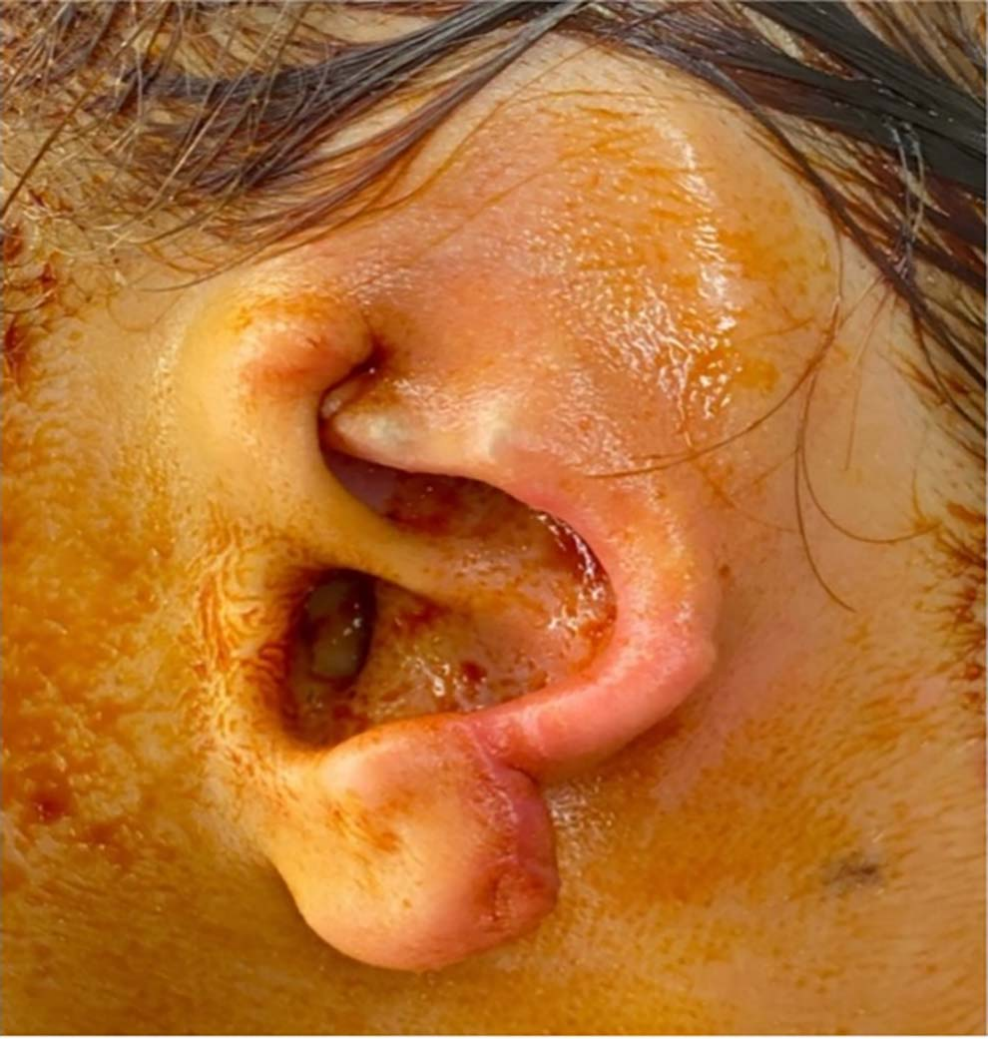
Anterior view of healed pocket burial.

The pocket healed without complications at 1 week postoperatively (Figure 6), and the second-stage procedure was planned for 1 month, with the anticipation of successful lamination of the buried ear cartilage.

**Figure 7.**
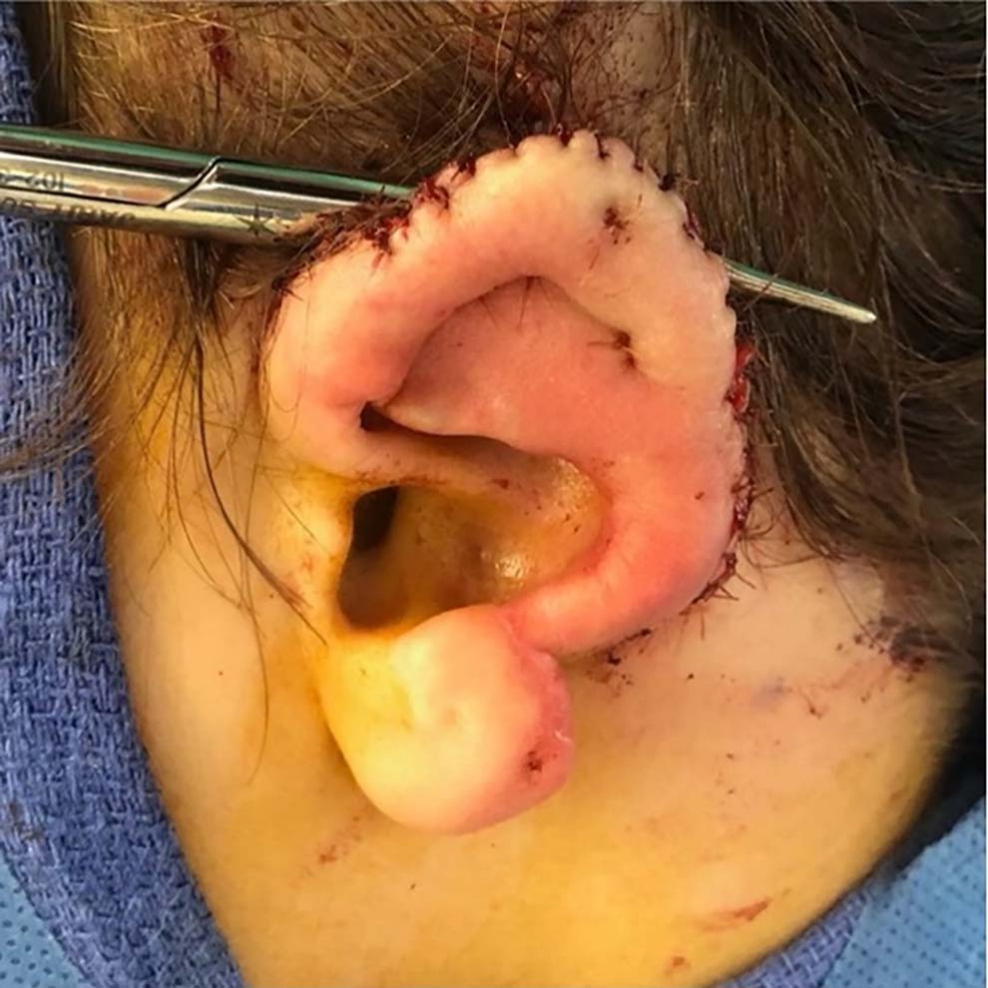
Anterior view of the helical rim showing suture wrapped around the edge of the helix.

At 29 days postoperatively, the second-stage procedure was performed. A subcutaneous dissection entering the plane deep to the laminated construct was performed, and successful construct elevation was achieved. To close the skin defect, the posterior auricular scalp was widely undermined, and a small back cut was made superiorly to diminish tension on the wound edges, allowing for rotation and advancement of the scalp flap to the level of the retroauricular sulcus. The flap was then secured to the base of the posterior auricular sulcus, leaving a 0.5-cm strip of the sulcus uncovered that was addressed with an 8 cm × 4 cm full-thickness skin graft obtained from the patient's left groin. The graft covered both the strip of posterior auricular sulcus and the posterior wall of the newly laminated construct. The anterior aspect of the helical rim skin was sutured in a manner to allow it to wrap around the edge of the helix so the anterior skin/skin graft junction scar would be positioned on the posterior aspect of the ear construct (Figure 7).

### Postoperative Results

**Figure 8.**
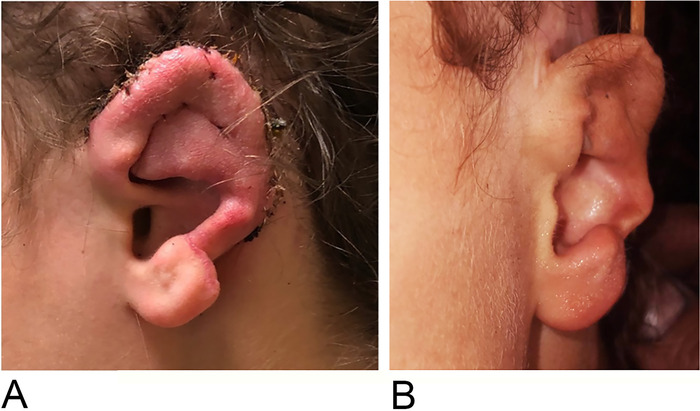
(A) Ear reconstruction at 2 weeks postoperatively. (B) Ear reconstruction at 3 months postoperatively.

The patient had no complications at 2 weeks postoperatively, and the auricular reconstruction was intact and displayed survival of the skin graft (Figure 8A). At approximately 3 months postoperatively, the patient continued to experience no postoperative complications and was aesthetically satisfied with the result (Figure 8B).

## DISCUSSION

Several methods of ear reconstruction have been described since 1920 when Gillies originally proposed the technique of retroauricular skin flaps,^15^ and Tanzer described the use of costal cartilage grafts in 1959.^16^ In modern practice, when surgeons are tasked with reconstruction of a total or near-total ear defect, the surgical mainstay is to use methods developed from microtia reconstruction.^17^ In 1993, Nagata described a 2-stage ear reconstruction without the use of skin grafts for total reconstruction of the auricle.^18^ In this technique, a semilunar costal cartilage is used for ear elevation, and a temporoparietal fascial flap is raised to provide circulatory support to the posterior segment. This method buries the reshaped costal cartilage graft beneath the native overlying skin of the hypoplastic outer ear and covers the 3-dimensional frame by using skin from the posterior ear lobe to resurface the concha.^18,19^

In 2004, Brunelli et al described the successful auricular reconstruction of a partially amputated ear in a patient following a dog bite; they performed a 3-stage technique using a costal cartilage autograft covered by a skin graft harvested from the mastoid region via use of a tissue expander.^5^ In 2008, Romo and Reitzen reported successful reconstruction with allopathic implants, bypassing the need for rib grafts and thus entirely removing the pain of donor site morbidity.^12^

In cases of traumatic amputation with the avulsed ear segment preserved, supermicrosurgery can be used for replantation. In 1966, Buncke and Shulz developed the microvascular anastomotic technique for total ear replantation in rabbits^20^ that established the technical details for the first successful human replantation reported by Pennington et al in 1980.^21^ If performed successfully, supermicroscopic ear replantation can result in natural- or near-natural–appearing replanted tissue; however, factors such as a crush injury, disrupted venous outflow, ischemic time, and extent of the amputation greatly affect tissue survivability.^22^ Since 1970, The Buncke Clinic has been a leading institution in advanced medical treatment using microsurgery. Founded by Harry J. Buncke, known as the Father of Microsurgery, the clinic has pioneered numerous advancements in the field of microsurgery.^23^ Worth noting, however, is that microscopic vessel grafting, while useful in cases of complex trauma, is associated with increased operative time, length of hospital stay, and postoperative complications, compared to less complex injuries for which successful replantation may be achieved using direct anastomosis.^24^

In cases of a large ear defect when the missing segment is unavailable for replantation, auricular reconstruction using a costochondral graft and pocket utilization is the preferred method.^25,26^ The costochondral construct makes a versatile graft and can be fashioned to form the desired shape of the outer ear.^23-28^ In the first stage of a 2-stage procedure, the autologous costal cartilage is buried for lamination in a skin pocket created in the posterior auricular space. In the second stage, the framework is elevated, and the posterior surface of the laminated ear construct is covered with a skin graft. We used the same technique in our case, except the patient's native avulsed ear cartilage was used in place of a rib graft. The patient did not experience postoperative complications and is happy with the results.

## CONCLUSION

In a traumatic avulsion of the outer ear with preserved native cartilage, a 2-stage technique of native cartilage lamination via posterior auricular pocket formation and skin graft placement can be an effective method to reconstruct the complex ear anatomy and may result in an aesthetically satisfactory outcome.

## Notes

*This article meets the Accreditation Council for Graduate Medical Education and the American Board of Medical Specialties Maintenance of Certification competencies for Patient Care and Medical Knowledge.*
